# Enhanced Spatial Transcriptomics Analysis of Mouse Lung Tissues Reveals Cell-Specific Gene Expression Changes Associated with Pulmonary Hypertension

**DOI:** 10.70322/jrbtm.2025.10004

**Published:** 2025-05-15

**Authors:** Hanqiu Zhao, Xiaokuang Ma, Peng Chen, Bin Liu, Jing Wei, John Zhang, Ankit A. Desai, Andrea L. Frump, Olga Rafikova, Michael B. Fallon, Shenfeng Qiu, Zhiyu Dai

**Affiliations:** 1 Department of Internal Medicine, University of Arizona College of Medicine-Phoenix, Phoenix, AZ 85004, USA;; 2 Division of Pulmonary, Critical Care Medicine, John T. Milliken Department of Medicine, Washington University School of Medicine in Saint Louis, Saint Louis, MO 63110, USA; 3 Basic Medical Sciences, University of Arizona College of Medicine-Phoenix, Phoenix, AZ 85004, USA;; 4 Department of Medicine, Indiana University Indianapolis, Indianapolis, IN 46202, USA;

**Keywords:** Spatial transcriptomics, Pulmonary hypertension, Fixed frozen tissue, Xenium platform, Endothelial cells, Arterialization, Mesenchymal transition

## Abstract

Spatial transcriptomics technologies have emerged as powerful tools for understanding cellular identity and function within the natural spatial context of tissues. Traditional transcriptomics techniques, such as bulk and single-cell RNA sequencing, lose this spatial information, which is critical for addressing many biological questions. Here, we present a protocol for high-resolution spatial transcriptomics using fixed frozen mouse lung sections mounted on 10X Genomics Xenium slides. This method integrates multiplexed fluorescent in situ hybridization (FISH) with high-throughput imaging to reveal the spatial distribution of mRNA molecules in lung tissue sections, allowing detailed analysis of gene expression changes in a mouse model of pulmonary hypertension (PH). We compared two tissue preparation methods, fixed frozen and fresh frozen, for compatibility with the Xenium platform. Our fixed frozen approach, utilizing a free-floating technique to mount thin lung sections onto Xenium slides at room temperature, preserved tissue integrity and maximized the imaging area, resulting in high-fidelity spatial transcriptomics data. Using a predesigned 379-gene mouse panel, we identified 40 major lung cell types. We detected key cellular changes in PH, including an increase in arterial endothelial cells (AECs) and fibroblasts, alongside a reduction in capillary endothelial cells (CAP1 and CAP2). Through differential gene expression analysis, we observed markers of endothelial-to-mesenchymal transition and fibroblast activation in PH lungs. High-resolution spatial mapping further confirmed increased arterialization in the distal microvasculature. These findings underscore the utility of spatial transcriptomics in preserving the native tissue architecture and enhancing our understanding of cellular heterogeneity in disease. Our protocol provides a reliable method for integrating spatial and transcriptomic data using fixed frozen lung tissues, offering significant potential for future studies in complex diseases such as PH.

## 1. Introduction

While bulk and single-cell transcriptomics technologies enable the quantification of the diverse mRNA transcripts that define cellular identity and function, they primarily analyze dissociated cells, thus losing the natural spatial context of molecules within cells and the spatial arrangement of cells within tissues or organs. Spatial context is crucial for addressing many biological questions, and spatially resolved genomic methods address these challenges by providing genome-scale measurements while preserving spatial information [1]. Therefore, revealing spatial information of cellular biomolecules is critically important, and understanding cellular functions is most meaningful within the native tissue context in which these cells reside.

Recently, advanced spatial transcriptomics methods have been developed to enable multiplexed mRNA in situ detection. Two main strategies are employed in spatial transcriptomics: multiplexed fluorescent in situ hybridization (FISH) techniques, such as MERFISH [2] and SeqFISH [3], and in situ capture followed by high-throughput sequencing, exemplified by Slide-seq [4]. A leading commercial provider of these technologies is 10X Genomics, offering platforms like Xenium (FISH- and imaging-based) and Visium/Visium HD (in situ sequencing-based) for spatial mRNA quantification. The Xenium platform, in particular, utilizes FISH to achieve high sensitivity and specificity for detecting individual RNA molecules, providing high spatial resolution for precise localization within tissue sections. To resolve gene expression changes on the Xenium platform, a panel of predesigned mRNA probes must be utilized. The platform offers multiplexing capability, allowing for the simultaneous detection of multiple RNA targets and enabling the study of several hundred to several thousand genes within the same tissue section. Additionally, Xenium provides quantitative measurements of RNA expression levels, which are essential for analyzing spatially heterogeneous tissues like the lung.

Here, we demonstrated high fidelity using ‘fixed frozen’ mouse lung sections on a Xenium platform. As a complete protocol for running tissue sections on the Xenium analyzer is beyond the scope of this article, we focused on the wet lab processes, including fixed frozen tissue preparation, cryosectioning, and mounting on Xenium slides. Our protocol enables mounting high-quality sections without tissue folding, preserving mRNA transcripts from degradation, while maximizing the use of the Xenium slide imaging areas to increase data output and reduce costs. Finally, we provided imaging and quantification data from a dataset that demonstrates the excellent results of this tissue preparation protocol and revealed differential gene expressions from a mouse model of pulmonary hypertension (PH).

## 2. Methods

### 2.1. Mice

*Egln1*^*f/f*^ (WT) and *Egln1*^*Tie2Cre*^ (KO) mice were generated as described previously [5]. Mice at the age of 2 to 3.5 months were used in these studies. All animal care and study protocols were reviewed and approved by the Institutional Animal Care and Use Committee of the University of Arizona.

### 2.2. Xenium Slide Thin Tissue Section Mounting

When the mice were under deep anesthesia, we opened the thoracic cavity to expose the heart and lungs. A 22-gauge perfusion needle was inserted into the left ventricle, connected to a 10 mL syringe pump filled with chilled 0. 01 M PBS, set to a flow rate of 2 mL min. After making a small incision in the right atrium using fine scissors, the pump was activated. Once the body was completely bloodless, we removed the syringe and replaced it with another 10 mL syringe containing chilled 4 paraformaldehyde. We then slowly and steadily perfused the lungs with 4% PFA for 5 min. Finally, the lungs were dissected and transferred to a 50 mL conical tube containing 30 mL of chilled 4% PFA for post-fixation. After 10–14 h, lung tissues were continued to be incubated in 30% sucrose (in 0.01 M PBS) for 48 h, until the tissues descended to the bottom of the container. Lung tissues were then embedded with OCT and immediately placed on dry ice. Wait until the OCT is completely frozen, turning white and hard. Then the samples were able to be sectioned in a cryostat or stored at −80 °C in a sealed container for long-term storage. The method was adopted from our prior work in brain tissue [6].

Embedded lung samples were cut into 10 μm thickness in a cryostat (Leica CM1950 model). Tissue sections were collected in a 6-well cell culture plate that contained pre-chilled 0.01 M PBS. The selected tissue sections were mounted on the Xenium slides under a stereo microscope (SZ16 model, Olympus, Tokyo, Japan) under the 1.0X objective. Repeat these floating section mounting steps until all imaging areas of the slide are mounted with sections. Carefully record and illustrate positions for each thin lung section after placement on the Xenium slide, including the metadata information for each section.

Xenium slides with samples were processed using a predesigned 379-gene mouse panel of probes on the Xenium Analyzer (10X Genomic) by the core facility at 10X Genomic. Tissue staining was performed using a combination of nucleus (DAPI), cellular content, and cell boundary markers with the Xenium Multi-Tissue Stain Mix (10X Genomics, PN 2000991), following guideline CG000749. This staining delineates cell boundaries and allows the segmentation of different cells throughout the tissue.

### 2.3. Xenium Data Analysis

Xenium data output was loaded on Xenium Explorer for general data visualization and analysis. For unsupervised clustering of the dataset, Xenium output data was processed using the Scanpy and Seurat packages. The LungMap Mouse Reference v.1.1 dataset was used for cell type label transfer and annotation. The Single Cell Proportion Test was performed using scProportionTest. The DEGs visualization was generated using scRNAtoolVis.

### 2.4. RNASCOPE and Immunofluorescent Staining

A Multiplex Fluorescent V2 RNAscope in situ hybridization (Advanced Cell Diagnostics, Newark, CA, USA) and immunostaining assay were conducted on lung cryosections obtained from mouse lung samples. The procedure involved fixing the tissue sections in 4% paraformaldehyde (PFA) at room temperature for 20 min, followed by blocking with 0.1% Triton X-100 in PBS containing 5% normal goat serum for 1 h at room temperature. The sections were then incubated with hydrogen peroxide at room temperature for 10 min, after which they were treated with Protease IV for 30 min at room temperature. The hybridization process included a mouse Ednrb (473801) probe for two hours at 40 °C, followed by a 30-min signal amplification using the RNAscope Multiplex Fluorescent v2 Assay (Advanced Cell Diagnostics), in accordance with the manufacturer’s guidelines. The signal development involved incubating the slides with HRP-C1 for 15 min, followed by Opal Dye 620 for 30 min. Finally, the slides were washed with PBS and incubated overnight at 4 °C with anti-CD31 (1:50, BD Bioscience, Warszawa, Poland, 550274) After three washes with 1× PBS, the sections were incubated for 1 h at room temperature with Alexa Fluor 488-conjugated anti-rat IgG (1:300, Thermo Fisher Scientific, Waltham, MA, USA, A21247). Nuclei were counterstained with mounting medium containing DAPI (SouthernBiotech, Birmingham, AL, USA, 0100–20), and images were captured.

### 2.5. Data and Code Availability

The xenium processed data were available at NCBI GEO dataset (GSE277936). Scripts used for analysis are available on GitHub (https://github.com/DaiZYlab/enhancedXenium (accessed on 5 January 2025)).

### 2.6. Statistical Analysis

Differential gene expression analyses between WT and KO samples for each cluster of interest were performed using the Wilcoxon Rank Sum Test algorithm with log normalized counts in the RNA assay as input. A threshold of 0.25 for log fold change, 0.05 for the adjusted *p*-value, and 0.1 for a minimal fraction of cells was applied for downstream analysis. A permutation test is used to calculate a *p*-value for each cluster, and a confidence interval for the magnitude difference is returned via bootstrapping. FDR < 0.05 was designated as significant.

## 3. Result

### 3.1. Fixed Frozen Mouse Lung Thin Section Mounting onto Xenium Slides

The fluorescent in situ (FISH) followed by imaging-based spatial transcriptomics studies require thin tissue sections mounted on the defined imaging area on a slide. The Xenium pipeline, offered by 10X Genomics, is a leading commercial platform for these studies. To obtain high-quality imaging data on the Xenium platform, sample preparation is critical. Currently, the Xenium platform is validated for formalin-fixed paraffin-embedded (FFPE) thin tissue sections [7], although fresh frozen tissue sections are also compatible with the recommended protocols [8]. However, many laboratories working with various tissue sections typically follow a protocol involving PFA fixation, cryoprotection, cryosectioning of thicker sections (e.g., 40 μm), followed by immunohistochemistry (e.g., with multichannel antibody labeling) or in situ hybridization for mRNA species (e.g., RNAscope). It is unclear whether this protocol is compatible with the recommended workflow from 10X Genomics.

**Figure 1.**
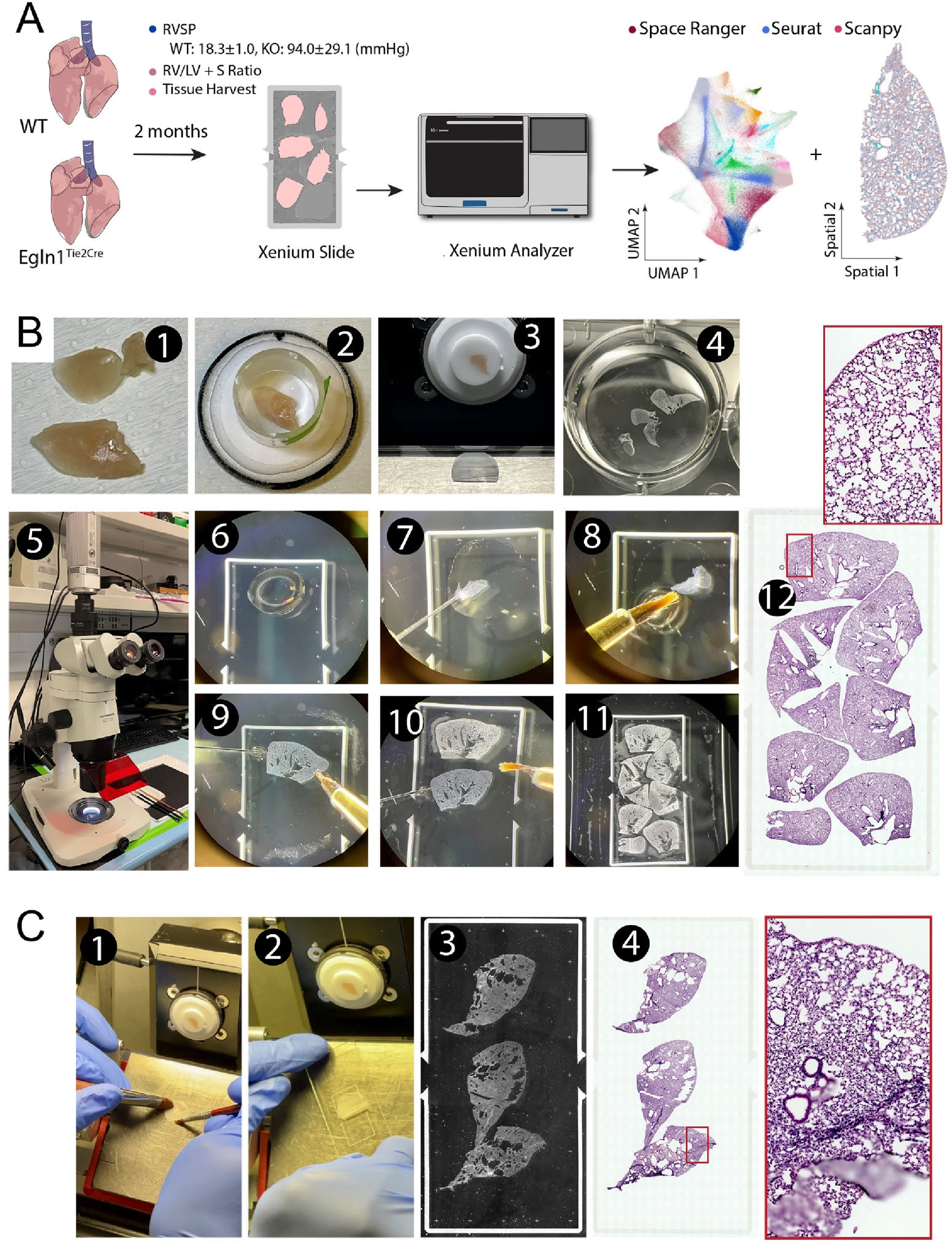
Protocol outline for fixed frozen mouse lung thin section mounting on a Xenium slide. (**A**) Protocol flowchart. Control (WT) and pulmonary hypertension (PH, *Egln1*^*Tie2cre*^, KO) models were generated and validated. Mouse lungs were collected after intracardial perfusion with 4% PFA. Thin cryosections were mounted on a Xenium slide and run on a Xenium analyzer with prebuild gene panels, followed by bioinformatics analyses. (**B**) Photomicrographs illustrate steps of mouse lung OCT embedding, thin cryosectioning and mounting on the Xenium slide. Tissue sections were further stained with HE to visualize tissue cytoarchitectures. (**C**) In comparison, the 10X Genomics validated fresh frozen tissue cryosectioning and mounting leads to tissue folding and insufficient utilization of the imaging area.

We tested our fixed frozen thin mouse lung sections for their compatibility on the Xenium based spatial transcriptomics (Figure 1A-protocol outline, Table 1). Briefly (Figure 1B), the Mouse lung was first fixed by 4% PFA through transcardial perfusion. It was then dissected out and further post-fixed in 4% PFA overnight at 4 °C. The lung was then cryoprotected, embedded in OCT compound, and sectioned on a cryostat into 10 μm thickness. To facilitate mounting on the Xenium slide imaging area, we adopted a free-floating approach under a dissection microscope, in which a small drop of nuclease free PBS was placed on a corner of the Xenium slide imaging area. A thin lung section was transferred to the PBS meniscus using a fire-polished glass probe. By alternating the glass probe and small trimmed paint brush, the thin section was able to be flattened and guided to the desired location inside the PBS. Once a tissue section was flattened, the paint brush was used to withdraw PBS and dry the section onto a Xenium slide. Another drop of PBS was then introduced in an adjacent area to enable mounting another section. Repeating this approach, most of the imaging area of the Xenium slide was efficiently covered. Once the Xenium slide was fully mounted with sections, the tissue section layouts were recorded into metadata information for each section (e.g., mouse, age, sex, genotypes, *etc*.). The slide was stored in a slide mailer at −80°C for up to three weeks before being analyzed on a Xenium Analyzer, which was typically operated by a core facility or a commercial service provider. In contrast to this fixed frozen protocol, we also mounted another set of mouse lung sections using the 10X Genomics- validated ‘fresh frozen’ method. Freshly dissected lungs were embedded in OCT and sectioned on a cryostat at a thickness of 10 μm. Efforts were made to minimize tissue rolling, and the thin sections were carefully picked up from the sectioning platform of a cryostat and directly placed onto the aligned Xenium slide imaging area. Care was also taken to prevent tissue overlapping. Despite multiple attempts, obtaining a completely flat, large tissue section proved to be challenging. Additionally, a much larger area was left empty to avoid section overlapping (Figure 1C).

### 3.2. Initial Analyses and Clustering of Lung Cells through Identified RNA Transcripts

Our previous studies employing single-cell RNA sequencing (scRNA-seq) and 10X Genomic Visium spatial transcriptomics analysis demonstrated that there was an increase in arterial endothelial cells (ECs) and a loss of distal capillary ECs in the lungs of *Egln1*^*Tie2Cre*^ mice, a severe pulmonary hypertension PH mice model with obstructive vascular remodeling [5,9]. Due to the limitation of losing spatial context and cell dissociation bias of scRNA-seq and the relatively lower resolution of Visium, we conducted a high-resolution Xenium in Situ Analysis using a predesigned 379-gene mouse panel of probes, which cover major cell types in the mouse tissue, to demonstrate the feasibility of using fixed frozen mouse sections with free-floating mounting onto Xenium slides (Figure 1A). The details of preparation of multiple fixed frozen and floating lung sections on Xenium slide were illustrated in Figure 1B and described in the Methods section.

**Figure 2.**
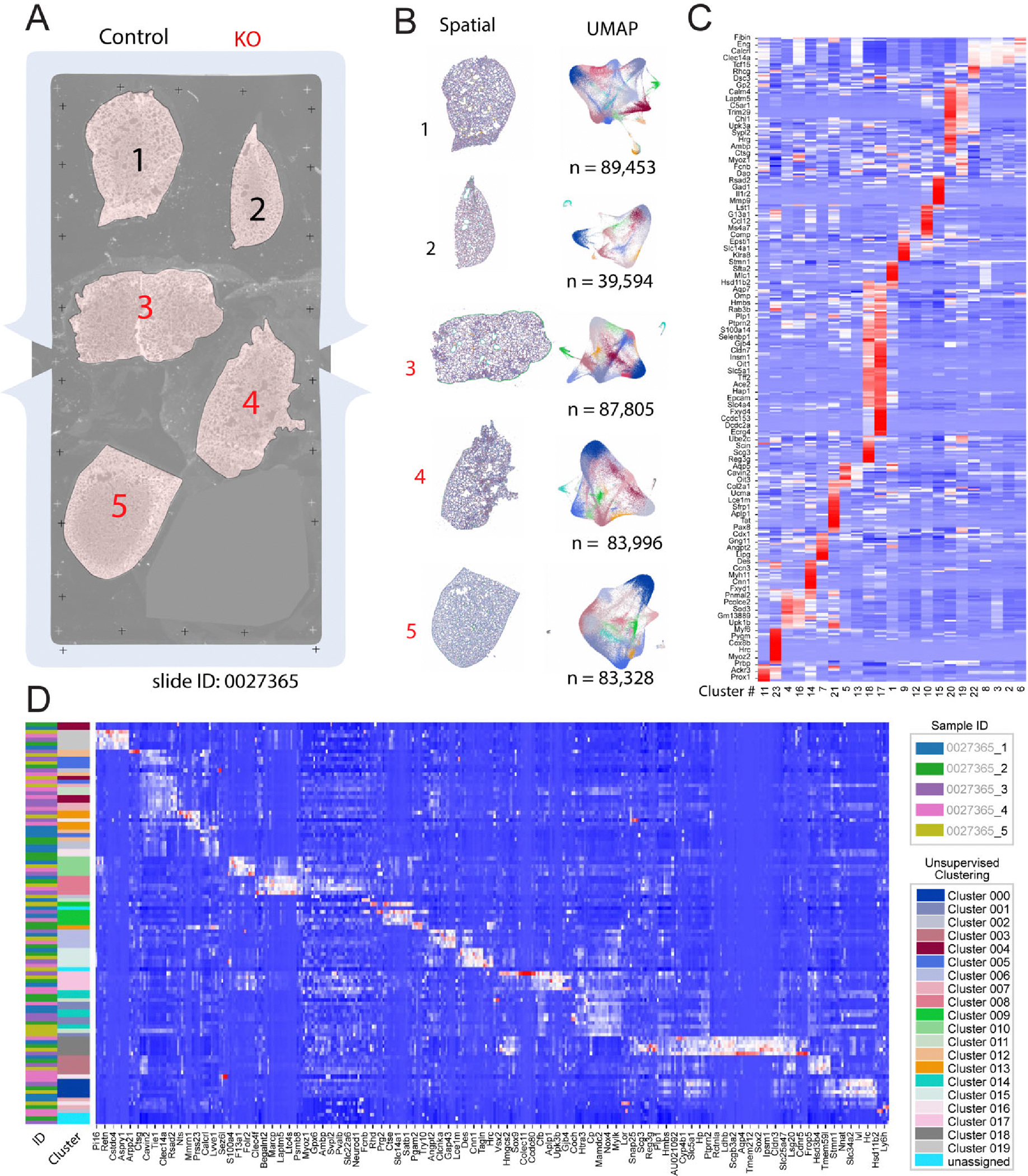
Initial analyses and clustering of lung samples. (**A**) Overview of tissue section layout on a Xenium slide. Five sections (two control and three *Egln1*^*Tie2cre*^/KO) were mounted in the imaging area. (**B**) Each tissue section was delivered as a separate dataset, which was plotted on both spatial coordinates (**left**) and UMAP embeddings (**right**). (**C**) Selected top gene markers from the 23 unsupervised clusters, with expression levels denoted by heatmap. The data was generated from 5 Xenium spatial data. (**D**) Heatmap of selected gene expressions further organized by sample ID and unsupervised Clusters. The data was generated from 5 Xenium spatial data.

We mounted 5 lung sections onto one Xenium slide imaging area (Figure 2A), of which three sections were from three *Egln1*^*Tie2Cre*^ (KO) mice, and two sections were from littermate controls. Xenium slides were probed and stained with cell segmentation kits, followed by processing on Xenium Analysis. We conducted further analysis using Seurat [10], Scanpy [11], and Squidpy [12] packages on the Xenium Analysis data output. Each tissue section was provided as a separate dataset, with cell segmentation already implemented on the Xenium analyzer. For each dataset, unsupervised clustering resulted in 23 clusters that were well separated into the spatial and UMAP space (Figure 2B). There was a high level of top marker gene expressions for each cluster, as shown by the heatmap (Figure 2C). After integration of the five samples, expression levels of the top marker genes for the 20 unsupervised clusters were plotted as an additional heatmap (Figure 2D, further sorted by sample ID and Clusters).

**Figure 3.**
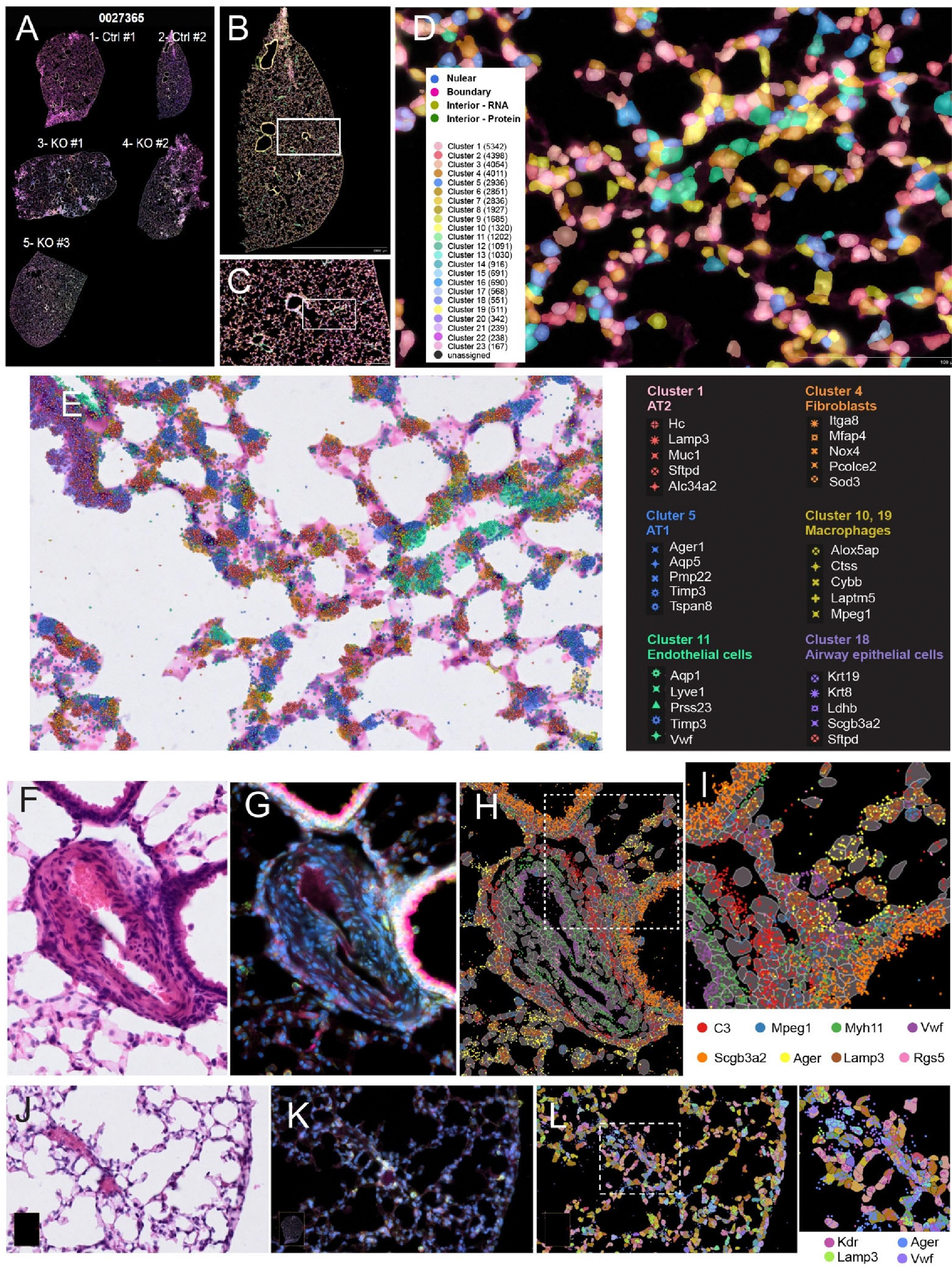
Cell staining based segmentation and spatial distribution of selected transcripts. (**A**) Overview of the 5 stained mouse lung thin tissue sections mounted on a Xenium slide. (**B**) Spatial plotting of detected cell clusters in one tissue section (Ctrl #2). (**C**) Enlarged view of the boxed area in (**B**). (**D**) Enlarged view of boxed area in (**C**), with detected cell types and boundaries overlaid on the stained tissue image. (**E**) The same field of view in (**D**), with detected transcript locations overlaid onto H & E-stained bright field image. Cell type annotation and their top gene markers were listed to the right. (**F**) Bright field view of a remodeling pulmonary artery from KO mice. (**G**) Fluorescent stain of the same region as in (**F**). (**H**) Cell segmentation and overlaid selected transcripts. (**I**) Enlarged view of boxed area in (**H**). (**J**) Bright field view of distal alveolars from WT mice. (**K**) Fluorescent stain of the same region as in (**J**). (**L**) Cell segmentation and overlaid selected transcripts of the same region in (**J**,**K**).

Next, we explored the spatial features of the tissue sections, including cell segmentation and the spatial embedding of selective transcripts. Cell segmentation utilized immunostaining and deep learning-based cell segmentation algorithms. Using a combination of nucleus (DAPI), cellular content, and cell boundary markers provided with the Xenium Multi-Tissue Stain Mix, the staining delineated cell boundaries, and imaging information was collected by the Xenium Analyzer instrument and processed to be segmented. Cell and tissue landmarks were further delineated through H & E staining. Figure 3A shows an overall staining of all five tissue sections acquired by the Xenium analyzer. In one control lung section (Figure 3B), unsupervised clustering revealed the spatial distribution of 23 clusters of cells. A selected area with vessel and alveolar space was further shown in Figure 3C. Figure 3D shows an enlarged view of a boxed region of Figure 3C, in which contours of segmented cells of different clusters (C1 through C23) were overlaid onto the stained tissue image. The locations and relative abundance of specific transcripts can be jointly visualized with nuclei staining and H&E staining at higher magnification. Figure 3E demonstrated an enlarged view of tissue section with detected cell types (“AT2”, “AT1”, “Fibroblasts”, “Macrophages”, “Endothelial cells”, “Airway epithelial cells”), with their spatial location of the top 5 marker gene transcripts overlaid on the H & E-stained tissue image. For example, cluster 1 expressed type 2 epithelial cell (AT2) markers *Lamp3, Muc1,* and *Sftpd*, and cluster 4 expressed fibroblast *markers Itga8, Mfap4, Nox4*, *etc*.

The combined staining protocol and high-resolution imaging enabled the visualization of detailed anatomical structures from selected regions of interest (ROIs). We further selected two ROIs representing severe pulmonary vascular remodeling from a KO dataset and distal vessels and alveolars from a WT dataset. Cell segmentation led to the identification of cell boundaries, and with that, the mRNA transcripts were able to be assigned to individual cells. Figure 3F (H & E-brightfield) and Figure 3G (fluorescent staining used for cell segmentation) showed an arterial vessel with severe vascular remodeling from KO mice. Figure 3J,K showed a distal vessel and alveolars from WT mice. Based on top marker gene transcripts, we identified multiple cell populations including large vessel ECs (*Vwf*), capillary ECs (Kdr), smooth muscle cells (*Myh11*), fibroblasts (*C3*), macrophages (*Mpeg1*), pericytes (*Rgs5*), airway epithelial cells (*Scgb3a2*), type 1 epithelial cell (*AT1*, *Ager*), and AT2 cells (*Lamp3*) (Figure 3H,I,L).

### 3.3. High Resolution Spatial Transcriptomics Revealed Major Cell Types in the Mouse Lung

**Figure 4.**
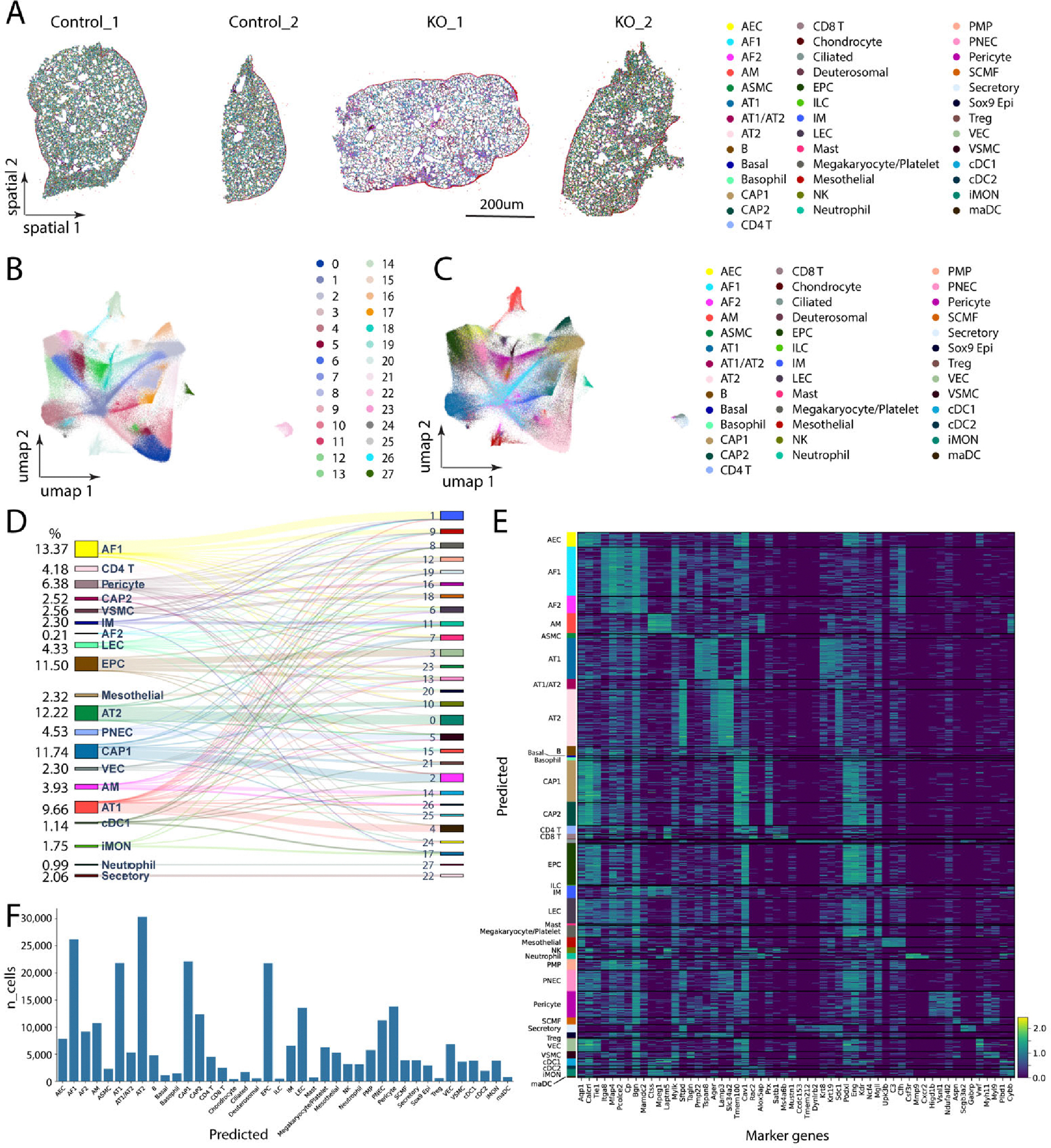
Customized analysis of Xenium dataset using Scanpy/Squidpy pipeline. (**A**) Spatial plot of four tissue samples with annotated lung cell types. (**B**) UMAP embedding of all the cells integrated after unsupervised clustering (*n* = 4). (**C**) Annotated cell types from the same UMAP space as in (**B**) (*n* = 4). (**D**) Sanky plot showing cell type transition from the 28 unsupervised clusters to the selected top 20 most abundant, annotated cell type clusters. Cells with less than 100 were excluded for Sanky analysis. (**E**) Heatmap revealed enriched top marker genes for each of the annotated cell types (*n* = 4). (**F**) Histogram distribution on the total number of 300,848 cells assigned to the predicted 40 annotated cell types (*n* = 4).

The data delivery deck from the Xenium analyzer can be visualized using Xenium Explorer, which also allows spatial coordinates of selected ROIs and cell metadata information to be exported for further customized analysis. The delivered data can readily be analyzed with the Scanpy and Squidpy pipelines, which allow cell label transfer when mapping to the LungMap Mouse Reference v1.1 dataset [13,14]. We then performed unsupervised clustering for the transcripts from 2 WT and 2 KO datasets and identified 28 clusters and predicted 40 lung cell types, including AT1, AT2, CAP1, CAP2, *etc*. (Figure 4A). Annotated cell types onto their spatial coordinates were plotted in Figure 4A. For the four samples (control_1, control_2, KO_1 and KO_2), the UMAP embedding of all the cells after unsupervised clustering was shown in Figure 4B. In addition, annotated cell types from the same UMAP space were shown in Figure 4C. We further plotted the cell type transition from the 28 unsupervised clusters to the selected 20 most abundant annotated cell type clusters using the Sanky plot (Figure 4D). Marker genes in these annotated cell types were shown in heatmaps (Figure 4E). The cell proportion analysis (including the total number of 300,848 cells assigned to the predicted 40 annotated cell types) showed that AT2, AT1, AF1, ECs (AEC, VEC, CAP1, CAP2, EPC), pericytes and LECs are relatively abundant compared to other cell types (Figure 4F). The cell proportions observed in spatial transcriptomics were relatively more similar to the observed in vivo cell proportions obtained from scRNA-seq analysis [15].

### 3.4. High Resolution Spatial Transcriptomics Identified Cell Type Specific Changes in the PH Lungs

**Figure 5.**
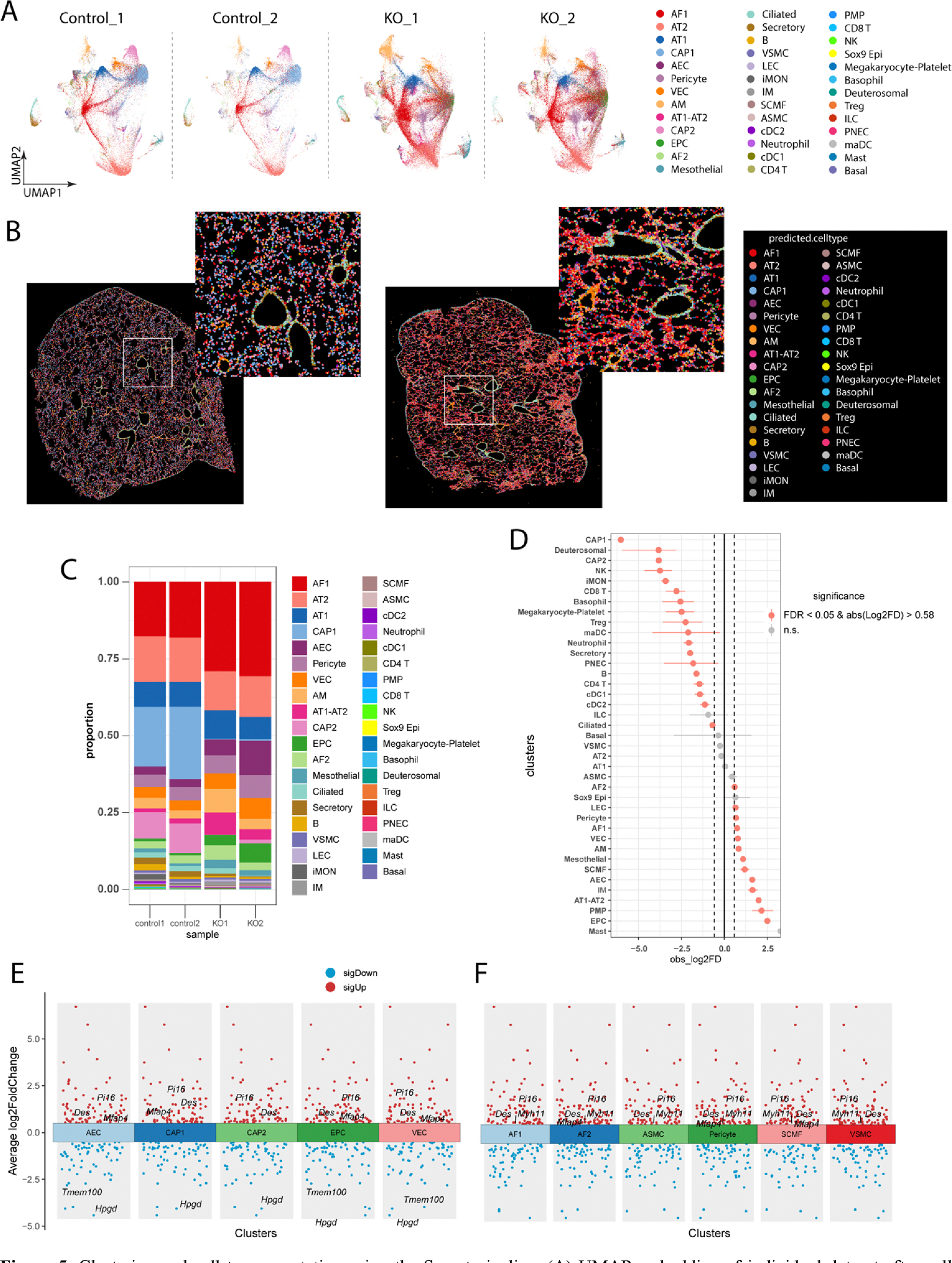
Clustering and cell type annotation using the Seurat pipeline. (**A**) UMAP embedding of individual dataset after cell annotation. (**B**) Spatial plot of WT and KO samples with annotated lung cell types. (**C**) Cell proportion analysis showed the increase of AF1, AF2, SCMF, AEC, EPC and a reduction of CAP1, CAP2, Treg, CD4 and CD8 T cells in KO mice (WT *n* = 2, KO *n* = 2). (**D**) Statistical analysis of the cell proportional change in KO mice. (WT *n* = 2, KO *n* = 2). (**E**,**F**) Differentially expressed genes (DEGs) on lung cell types between WT and KO mice. (WT *n* = 2, KO *n* = 2).

Next, we performed the Seurat V5 pipeline to further compare the cell type-specific changes, including the cell proportion change and differential gene expression (DEG) analysis between control and KO mice. Similar cell type annotation and spatial features were observed between Scanpy and Seurat analysis using UMAP and Spatial Plots (Figure 5A,B). We then evaluated the cell proportion change between control and KO mice. We found that many cell types were increased or decreased in the PH mice. For example, EC subpopulations such as EPC, AEC and VECs, macrophage subpopulations such as alveolar macrophages (AM) and interstitial macrophages (IM), fibroblasts subpopulations AF1 and SCMF, pericytes, and lymphatic ECs were increased in the KO mice, whereas CAP1, CAP2, T cells were reduced in the KO mice compared to WT mice (Figure 5C,D). We then performed the cell type specific DEGs analysis between control and KO mice. We found that mesenchymal markers, including *Des*, *Mfap4*, *Pi16* were upregulated in the EC subpopulations (AEC, CAP1, CAP2, and VEC) from KO mice compared to control (Figure 5E), suggesting that ECs underwent endothelial mesenchymal transition in PH. Moreover, we also observed the increase of myofibroblast markers *Myh11* in the KO fibroblast populations (AF1, AF2, SCMF) compared to WT (Figure 5F), indicating the activation of myofibroblasts in the KO lungs.

### 3.5. Increased Arterialization in Distal Microvasculature in PH by High Resolution Spatial Transcriptomics

**Figure 6.**
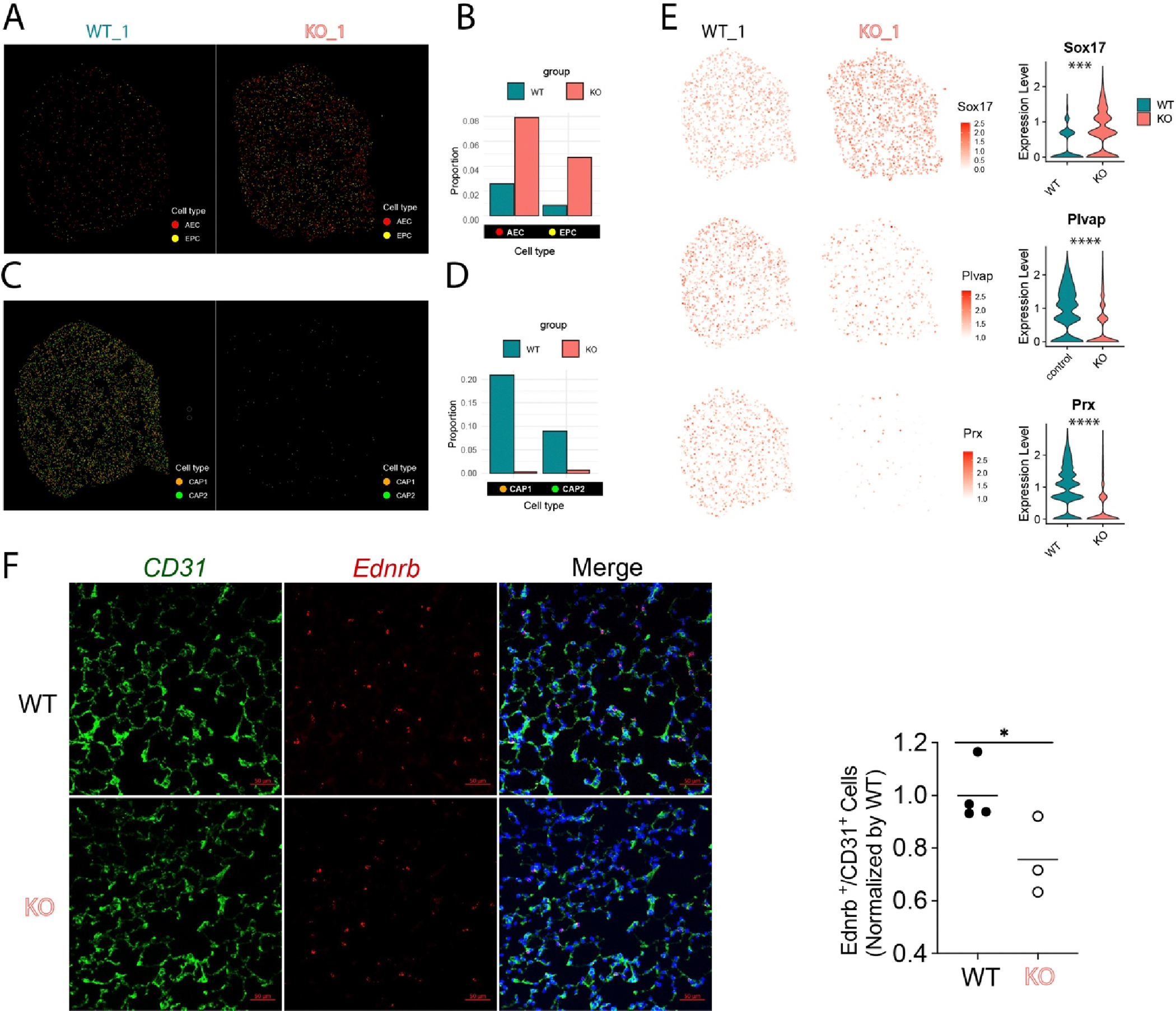
Spatial plot showing the change of EC subpopulation in PH mice. (**A**) Spatial plots showing the arterial ECs (AEC) and endothelial progenitor cells (EPC) were increased in KO mice (*n* = 2). (**B**) Quantification of AECs and EPCs cell proportions in (**A**). (**C**) Spatial plots showing the general capillary ECs (CAP1) and aerocytes (CAP2) were reduced in KO mice (*n* = 2). (**D**) Quantification data of AECs and EPCs in (**C**). (**E**) Spatial and Violin plots showing the increased AEC marker *Sox17*, decreased CAP1 (gCap) marker *Plvap*, and decreased CAP2 (aCap) marker *Prx* in the KO mice (*n* = 2). *** *p* < 0.001, **** *p* < 0.0001. (**F**) Immunostaining and RNASCOPE analysis validated the reduction of CAP2 (*Ednrb*^+^/CD31^+^) in KO mice (WT *n* = 4, KO *n* = 3). * *p* < 0.05.

Our recent studies demonstrated that there was an increase of arterialization in the distal lung in the PH using scRNA-seq and Visium studies [9,16]. We further extracted the ECs from the Xenium data and confirmed that there is a loss of capillary ECs (including CAP1 and CAP2) and an increase in arterial EC (AEC) and endothelial progenitor cells (EPCs) [13] in PH (Figure 6A). Spatial mapping also demonstrated that the AECs and EPCs were increased in the distal microvasculature in the KO lungs compared to WT lungs (Figure 6B). We also plotted the individual markers for AEC, CAP1 and CAP2, and we found that AEC marker *Sox17* was significantly increased (*** *p* < 0.001, Wilcoxon test), whereas CAP1 marker *Plvap* and CAP2 marker *Prx* were both reduced in the KO lungs (**** *p* < 0.0001, Wilcoxon test, Figure 6E). Immunostaining and RNASCOPE analysis further validated that CAP2 cells labeled by CD31 and *Ednrb* were significantly reduced in the KO lungs (Figure 6F).

## 4. Discussion

In summary, the results from this experiment demonstrated the feasibility of using fixed frozen mouse lung sections with free-floating mounting onto Xenium slides for the spatial transcriptomics studies. The integration of spatial and gene expression data through these analyses provided a comprehensive view of the cellular architecture and the molecular landscape within the mouse lung. This detailed classification and mapping of cell types enhances our understanding of the cellular responses to various physiologic and pathologic conditions, such as PH, and facilitates studies of cell-cell interactions and along with their functions within the native tissue context.

Our data obtained ~0.3 million cells and 1.3 million transcripts from 5 mouse lung tissues. We identified 40 major cell types in the lungs based on the limited set of genes available in the mouse multi-tissue panel provided by 10X Genomics. The results revealed abnormal changes of cell proportions in the PH-conditioned mice, such as an increase of AECs, AM and a reduction of CAP1 and CAP2. Our data also identified DEGs across multiple cell types of PH-exposed mice, such as an increase of mesenchymal markers in the EC subpopulations in PH mice. Further studies, including human sample validation, are warranted to confirm the generality of our findings.

In addition to the technical complexity, high cost, and limited gene panel, another challenge for Xenium platform analysis is tissue section preparation. High-quality sample preparation is crucial for obtaining FISH-imaging data on the Xenium platform. To date, the platform is optimized for formalin-fixed paraffin-embedded (FFPE) thin tissue sections, though fresh frozen tissue sections are also compatible with the recommended protocols. In many laboratories, tissue section preparation involves PFA fixation, cryo-protection, cryosectioning immunohistochemistry staining or in situ hybridization often uses thicker sections. Whether this latter protocol for sectioning is compatible with the 10X Genomics recommended Xenium workflow remains unknown. Our floating mounting technique for ‘fixed frozen’ thin lung sections on Xenium slides enables mounting at ambient temperature while preserving mRNA integrity for multiple rounds of high-plex hybridization of probes. A significant advantage of our method is the ability to mount sections in a drop of PBS under a dissection microscope using very ultrathin-tipped, rigid tools (e.g., pulled glass probes). The section can be easily moved to different locations on the slide by guiding the PBS volume to a specific location. This ensures maximum utilization of the imaging space, thereby increasing the transcriptomic yield and significantly reducing experimental costs. We envision that this protocol will be compatible with the latest Xenium Prime 5K panel. In addition, due to the large imaging area of the Xenium slide (10.45 mm × 22.45 mm), thin sections from multiple tissue types from the same species (e.g., brain, lung, kidney, *etc*.) can be mounted on the same slide. This would significantly lower down the cost, and enable increased detection of multiple biological replicates, and multiple experiments/projects to be completed from one experiment.

### Limitations of This Study

There are some limitations to our proposed method. Our current study used *n* = 2 for WT and *n* = 2 for KO mice for the spatial analyses, which may underpower statistical comparisons. Although the resolution of Xenium technology is subcellular, due to the limitation of imaging and cell segmentation methods, for the structure of the distal area of the lung, “single cell” may not be easily segmented, especially the microvessels between alveolars. Secondly, the available mouse gene panel used in this study included only 379 genes, with an even smaller subset being lung-specific. Although this restricted panel enables spatially resolved gene expression analysis, it covers only a small fraction of the mouse transcriptome, potentially introducing bias in gene expression and pathway analyses toward the included genes. As a result, novel signaling pathways, particularly those involving lowly expressed or uncharacterized genes, may be underrepresented or entirely missed. A larger set of DEGs may result from a gene panel with larger coverage, such as the latest 5K panel from 10X Genomics or using probes for whole transcriptome coverage. The protocol as presented may require fine-tuning and validation for other tissues based on their morphology, thickness, and fixation protocols. In addition, multiple practice rounds may be needed to achieve proficient mounting under the microscope and to maximize the usage of the imaging area and data gain on the Xenium slide.

## Figures and Tables

**Table 1.** Key resources table.

Reagent or Resource	Source	Identifier
**Experimental models: Organism**		
Mouse: C57BL/6J 2–3.5 months, *Egln1^f/f^* (WT), *Egln1^Tie2Cre^* (KO)	Ref. [5]	
**Chemicals**		
1X PBS	Gibco	10010-023
Paraformaldehyde	Sigma (Kanagawa, Japan)	158127
Sucrose	Sigma	S9378
O.C.T. compound	SAKURA (Osaka, Japan)	4583
H&E Staining Kit	Abcam (Cambridge, UK)	ab245880
Glycerol	Sigma	G5516
100% Ethanol	Sigma	E7023
Goat serum	Cell-Signaling Technology (Danvers, MA, USA)	5425S
Triton^™^ X-100	Sigma	No. 9036-19-5
anti-CD31	BD Bioscience	550274
Mouse Ednrb probe	Advanced Cell Diagnostics	473801
Alexa Fluor 488-conjugated anti-rat IgG	Thermo Fisher Scientific	A11006
DAPI	SouthernBiotech	0100-20
**Other**		
Xenium Slides & Sample Prep Reagents	10X Genomics (Pleasanton, CA, USA)	PN-1000460
Xenium Decoding Consumables	10X Genomics	PN-1000487
Xenium Mouse Tissue Atlassing Panel	10X Genomics	PN-1000627
Xenium Cell Segmentation Add-on Kit (2 rxns)	10X Genomics	PN-1000662
Stereo microscope	Olympus (Tokyo, Japan)	SZX16
Cryostat	Leica (Wetzlar, Germany)	CM1590
Xenium Analyzer	10X Genomics	PN-1000569
**Software**		
Xenium Analyzer	10X Genomics	Version 3.1
R	Opensource	R-Ver 4.3
RStudio	Opensource	Released 2023.03.02

## Data Availability

The xenium processed data were available at NCBI GEO dataset (GSE277936). Scripts used for analysis are available on GitHub (https://github.com/DaiZYlab/enhancedXenium (accessed on 5 January 2025)). Other data, analytical methods, and materials that support the findings of this study will be available to other researchers from the corresponding authors on reasonable request.
